# Correlates of Discordance between Perceived and Objective Distances to Local Fruit and Vegetable Retailers

**DOI:** 10.3390/ijerph16071262

**Published:** 2019-04-09

**Authors:** Katherine L. Baldock, Catherine Paquet, Natasha J. Howard, Neil T. Coffee, Anne W. Taylor, Mark Daniel

**Affiliations:** 1 Australian Centre for Precision Health, School of Health Sciences, University of South Australia, Adelaide, SA 5001, Australia; catherine.paquet@unisa.edu.au; 2 Division of Health Sciences, University of South Australia, Adelaide, SA 5001, Australia; natasha.howard@sahmri.com; 3 Wardliparingga Aboriginal Health Equity Theme, South Australian Health and Medical Research Institute, Adelaide, SA 5001, Australia; 4 Centre for Research & Action in Public Health, Health Research Institute, Faculty of Health, University of Canberra, Canberra, ATC 2601, Australia; neil.coffee@canberra.edu.au (N.T.C.); mark.daniel@canberra.edu.au (M.D.); 5 Population Research and Outcome Studies, Discipline of Medicine, The University of Adelaide, Adelaide, SA 5001, Australia; Anne.taylor@adelaide.edu.au; 6 South Australian Health and Medical Research Institute, Adelaide, SA 5001, Australia; 7 Department of Medicine, St Vincent’s Hospital, The University of Melbourne, Melbourne, VIC 3065, Australia

**Keywords:** perceptions, geographic information system, neighbourhood, walkability, food environment, Australia

## Abstract

*Background*: Perceptions of neighbourhood attributes such as proximity of food retailers that are discordant with objective measures of the same are associated with poor health behaviours and weight gain. Factors associated with discordant perceptions are likely relevant to planning more effective interventions to improve health. *Purpose*: Analysis of cross-sectional relationships between individual and neighbourhood factors and overestimations of walking distances to local fruit/vegetable retailers (FVR). *Methods*: Perceived walking times, converted to distances, between participant residences and FVR were compared with objectively-assessed road network distances calculated with a Geographic Information System for *n* = 1305 adults residing in Adelaide, South Australia. Differences between perceived and objective distances were expressed as ‘overestimated’ distances and were analysed relative to perceptions consistent with objective distances. Cross-sectional associations were evaluated between individual socio-demographic, health, and area-level characteristics and overestimated distances to FVR using multilevel logistic regression. *Results*: Agreement between objective and perceived distances between participants’ residence and the nearest FVR was only fair (weighted kappa = 0.22). Overestimated distances to FVR were positively associated with mental well-being, and were negatively associated with household income, physical functioning, sense of community, and objective distances to greengrocers. *Conclusions*: Individual characteristics and features of neighbourhoods were related to overestimated distances to FVR. Sense of connectivity and shared identity may shape more accurate understandings of local resource access, and offer a focal point for tailored public health initiatives that bring people together to achieve improved health behaviour.

## 1. Introduction

The impact of the neighbourhood built environment on residents’ behaviour and health outcomes is dependent upon how residents perceive and engage in their local environment [1,2]. It is therefore important to understand the influence of both resident perceptions and objective assessments of neighbourhood attributes on health and behaviour. Considerable evidence has demonstrated that perceived and objective measures of the same neighbourhood construct have at best low to moderate correspondence [3,4,5,6,7,8,9,10,11,12], suggesting that objective and perceptual measures of neighbourhood features, including the food environment [13], are not necessarily equivalent.

Previous research demonstrates that the discordance between perceived and objective measures of the neighbourhood food environment has relevance for health behaviours and outcomes. For instance, individuals living within one kilometre of a supermarket who did not perceive a supermarket within walking distance from home have been found to consume fewer fruits and vegetables than those with accurate perceptions of supermarket access [14]. In a more recent study, overestimating distances from one’s home to the nearest fruit and vegetable retailer or public open space was associated with measured cardiometabolic risk outcomes [15]. Gaining insight into the factors associated with discordant perceptions of the neighbourhood food environment may improve our understanding of the factors influencing resource utilisation decision-making and food behaviour, and subsequent health outcomes. Such knowledge could also inform interventions to improve neighbourhood perceptions [9,16,17], and would enable targeting of interventions to improve resident perceptions, in multi-level interventions targeting individuals and community features. Additionally, such information could be beneficial for improving statistical models evaluating associations between the neighbourhood food environment and health to include potential confounders where only perceived measures of environment are available [16].

The factors associated with concordance and discordance between perceived and objective measures of environments have been examined in just a few studies to date, primarily in relation to the physical activity environment. For example, individual socio-demographic factors such as age, gender and household income have been associated with both the concordance [16,18] and discordance [9,19] between perceived and objective measures of the physical activity environment. Concordance between perceived and objective measures of the physical activity environment has been associated with greater levels of physical activity [8,18] and lower body weight [18], whereas discordance has been associated with less physical activity [9,16] and greater body weight [9]. Accurate perceptions of the physical activity environment have also been associated with high social cohesion, a greater number of parks, and number of amenities in the nearest park [18]. Given the relevance of misperceptions of the food environment to health and behaviour [14,15], it is important to examine individual and area-level factors relating to discordant perceptions of the food environment.

Other individual-level factors that have been examined to a limited extent may also be relevant predictors of inaccurate perceptions of the neighbourhood environment. For example, evidence suggests that one’s emotional state, a component of mental well-being [20], can influence perceptual and evaluative judgements [21] or cognition [22], whereby a positive effect is associated with greater use of heuristics in evaluation and decision making [23]. Physical attributes of individuals may also impact on perceptions of environment, for example those who have to exert more effort perceive destinations as further away [24], distances appear greater to unfit people compared to fit people [25], and people who weigh more perceive distances as greater [26]. Reduced physical abilities or increased physical exertion may contribute to a discordance between perceived and objective measures of distance due to lesser opportunities to acquire specific knowledge about the neighbourhood environment such as destinations and routes through active transport or recreational walking in the neighbourhood. It has further been argued that motivation to utilise particular neighbourhood resources can shape perceptions [27]. Certain neighbourhood contexts may also contribute to the mismatch between specific perceived and objective neighbourhood attributes.

Negative evaluations of the overall quality of the neighbourhood may translate into a perceived-objective mismatch of the health-promoting nature of neighbourhoods in order to reduce cognitive dissonance, the psychological discomfort caused by inconsistent opinions, attitudes and behaviours [28]. For instance, perceiving a lack of locally available and accessible neighbourhood resources may correlate with perceiving the neighbourhood as unsafe, ensuring (even if false) a consistent perspective to reduce cognitive dissonance. Additionally, local access to places where the community can meet and interact, such as the retail environment or in parks and other open spaces, can provide opportunities for community connections to be met [29]. Having a greater sense of community may be related, then, to the use of local resources, which contributes to greater local knowledge and a lower likelihood of perceiving the neighbourhood environment differently from the objectively measured reality. Moreover, some evidence suggests that longer actual distances to local destinations are more likely to be underestimated [3]. Despite their relevance to perceptions that are discordant with objective environment measures, these factors have not, to date, been examined as potential predictors of inaccurate perceptions.

The first aim of this study was to assess the agreement between objective and perceived measures of distance to a health-promoting neighbourhood resource, namely fruit and vegetable retailers (FVR). The second aim was to evaluate a range of individual-level socio-demographic, behavioural, physical functioning and mental well-being characteristics, as well as perceptions of the neighbourhood social context and objective distance to resources in relation to the overestimated distances to FVR.

## 2. Methods

### 2.1. Sample

This study was conducted as part of the Place and Metabolic Syndrome (PAMS) project, drawing on individual data from the North West Adelaide Health Study (NWAHS). The NWAHS is a longitudinal cohort study for which three waves of data collection have been undertaken to date. The baseline sample of 4056 randomly selected adults aged 18 years and over was originally recruited between 2000 and 2003 from the northern and western metropolitan regions of Adelaide, Australia [30,31]. Detailed methods for the NWAHS have been described previously [30,31]. In 2006, approximately 1.1 million persons resided in the Adelaide metropolitan region, a coastal Australian city which extends 30 km east–west, and 80 km north–south [32]. NWAHS data collected across Wave 2 (2004–2007) were utilised for this cross-sectional analysis, as this was the only Wave at which all required measures were available. Health behaviours, physical functioning, mental well-being, and socio-demographic information were self-reported at the Wave 2 recruitment telephone interview. Neighbourhood sense of community was collected from participants during a telephone interview, and perceptions of neighbourhood attributes were elicited via a postal or online survey conducted after the Wave 2 clinic visit. All participants with a valid residential address were assigned a geo-reference corresponding to their place of residence to enable individual data to be joined with built environment data. The PAMS project was approved by the Human Research Ethics Committees of the Central Northern Adelaide Health Service (Application No.: 2010010) University of South Australia (Protocol No.: P029/10), and South Australian Department of Health (Protocol No.: 354/03/2013).

### 2.2. Measures

#### 2.2.1. Outcome Measure: Overestimated Distance to Nearest FVR

Overestimations of the distances to the nearest FVR were derived from perceived and objectively-assessed measures of distance between each participant’s residence and their nearest FVR.

Participants reported perceived distance (walking time) from their residence to the nearest FVR via two questions in the land-use mix diversity subscale of the Australian version of the Neighbourhood Environment Walkability Scale (NEWS-AU) [33], a modified version of the NEWS [34]. Perceived walking time to the nearest of either a supermarket or greengrocer (collectively defined as FVR) was classified into five categories, including: (1) 1–5 min, (2) 6–10 min, (3) 11–20 min, (4) 21–30 min, and (5) more than 30 min.

Arc GIS 9.3 software (Environmental Systems Research Institute, Redlands, CA, USA) was used to select all FVR within the Adelaide metropolitan region using spatial location information obtained from the 2007 South Australian Retail Database (RDB) [35]. FVR were defined as supermarkets and greengrocers in order to match the NEWS-AU items. Distances to FVR were derived from road network distances from geo-referenced participant residences to the nearest FVR, calculated using the ArcGIS 9.3 Network Analyst tool. Distance categories were interpreted in walking time (minutes) to correspond to the perceived walking times based on a moderate adult walking speed of 4.8 km (3.0 miles) per hour [36]. Actual distances to FVR were categorised to correspond to the perceived distance categories for FVR as follows: (1) 0–400 m (1–5 min walk), (2) 401–800 m (6–10 min walk), (3) 801–1600 m (11–20 min walk), (4) 1601–2400 m (21–30 min walk), and (5) greater than 2400 m (greater than a 30 min walk).

To derive overestimations of distance to the nearest FVR, a difference score was first calculated between perceptions of distance and objective distance by subtracting the perceived categorical walking time from the objective categorical walking time. Negative difference scores indicated that the perceived distance was greater than the actual distance (i.e., overestimated distances). This analysis concentrated on negative difference scores (overestimated distances) compared to perceptions consistent with objective distances. From the difference score, a dichotomous variable was calculated indicating whether resident perceptions of distance to the nearest FVR from home were overestimated or consistent with the objective distance category.

#### 2.2.2. Potential Correlates of Overestimated Distances to FVR and POS

Age (in years), gender, educational attainment assessed as less than bachelor’s degree or bachelor’s degree or higher, household income (AUD$) assessed as $20,000 or less, $20,001 to $60,000, or greater than $60,000, and the length of time lived in the current residence were included as socio-demographic factors.

Fruit and vegetable intake was self-reported via two questionnaire items previously used in the Australian National Health Survey [37], expressed as the total number of serves of fruits and vegetables usually consumed each day.

Physical activity was assessed via several questions also derived from the Australian National Health Survey [37], which asked respondents to report the frequency and duration of walking, moderate activity and vigorous activity they had undertaken for fitness, recreation or sport over the previous two weeks. From these questions, a measure of total physical activity was calculated by multiplying the number of times physical activity had been undertaken in the last two weeks by the average time per activity session by the intensity of the session, where intensity, or metabolic equivalent of task (MET), was estimated for each of the three categories of exercise identified in the survey, as follows: 3.5 for walking; 5.0 for moderate exercise; and 7.5 for vigorous exercise [37].

The generic Medical Outcomes Trust Short Form 36 (SF-36) version 1 Physical Functioning and Mental Health subscales [38] was used as measures of physical functioning and mental well-being. The two subscales were transformed to a scale of 0–100.

Neighbourhood perceptions included sense of community and perceived crime in the local area. Participants were asked how strongly they agreed with the statement: “I feel a sense of community with others in my local neighbourhood”, rated on a five-point scale (strongly disagree (0) to strongly agree (4)). Perceived crime in the neighbourhood was operationalised using six items from the NEWS-AU. In a factor analysis undertaken on the NEWS-AU items in a previous study [39], six items were found to load on a *crime* factor (Cronbach’s alpha = 0.80). The *crime* factor expressed responses to perceptions of crime in the local area, for example, “there is a lot of petty crime in my local area (e.g., vandalism, shoplifting)”, and “the level of crime in my local area makes it unsafe to walk at night”. The standardised factor score was used to represent perceived crime in the local area. A higher score indicates a higher level of perceived crime.

Area SES was included in models because the variables representing overestimated distances are dependent on objective measures of resource access and availability. Resource distribution across local areas has been related to area socioeconomic conditions [40]. State Suburb-level median household income (AUD) and educational attainment expressed as the proportion of the State Suburb with a Bachelor’s degree or higher were obtained from the 2006 Australian Bureau of Statistics Census of Population and Housing [41] and ascribed to participants based on their residential address. State Suburbs are a derived Census Geographic Unit which are formed by aggregating the finest Census unit to align with the most recent gazetted suburb at the time of the Census [42].

### 2.3. Data Analysis

The level of agreement between objectively-assessed and perceived distance to FVR was assessed by weighted kappa, and interpreted according to the criteria of Landis and Koch [43], where kappa 0.00–0.20 = slight; 0.21–0.40 = fair; 0.41–0.60 = moderate; 0.61–0.80 = substantial; and 0.81–1.00 = almost perfect. Kappa values were aggregated using a weighted average, using kappa’s precision (1/variance) as weights [44]. Multilevel logistic regression models with a random State Suburb-level intercept were used to evaluate associations between overestimated distances to FVR and age, gender, educational attainment, household income, length of time in current residence, fruit and vegetable intake, physical activity level, physical functioning, mental well-being, sense of community, perceived crime, area-level income, and objective distances to FVR, accounting for the clustering of participants within State Suburbs. All potential correlates were entered simultaneously into a multivariable model. All analyses were conducted using SAS (version 9.3; SAS Institute Inc., Cary, NC, USA). Statistical significance for all models was considered at α = 0.05.

## 3. Results

The analytic sample for this study (*n* = 1305) included NWAHS participants with complete data for all measures. Of the *n* = 3205 participants at Wave 2 of the NWAHS, *n* = 1943 completed the neighbourhood perceptions questionnaire. Of those, participants who were not residing within the Adelaide metropolitan region at Wave 2 (*n* = 87), who did not have a valid address for geocoding (*n* = 26), and who moved residences between the Wave 2 clinic visit and the follow-up telephone interview (*n* = 124) were excluded from analyses. Also excluded were participants with missing data for any of the variables included in analyses, including perceived environment measures: *n* = 268; socio-demographic factors: *n* = 29; self-reported health and health behaviours: *n* = 104. The 1305 participants were clustered within *n* = 182 State Suburbs within metropolitan Adelaide. Table 1 presents the individual and area-level characteristics of the analytic sample.

The level of agreement between perceived and objectively-assessed distances to the nearest FVR, indicated by weighted kappa coefficients, is presented in Table 2. Agreement between objective and perceived distances for each of the component measures of FVR and the overall classification was slight to fair.

Overestimating the distance to the nearest FVR was positively associated with mental well-being, and negatively associated with household income, physical functioning, sense of community, and the objective distance to the nearest greengrocer (Table 3).

## 4. Discussion

Building on the emerging body of research examining correlates of the discordance between perceived and objective measures of health-promoting neighbourhood attributes, this study yielded novel findings indicating that socio-demographic, physical functioning, and mental well-being characteristics, as well as residents’ sense of community and the objective distance to resources were associated with the overestimation of distance to FVR.

The correspondence between perceived and objective access to FVR in the present study was slight to moderate, consistent with the previous limited research on this topic. Low to moderate correspondence, assessed using a variety of methods, has been observed between perceived and objective access to supermarkets in four previous studies [3,7,8,12].

The present study found that those with a greater sense of community were less likely to have misperceived the distance to the nearest FVR. This finding is similar to that of Lackey and colleagues [18], who found that the perception of high neighbourhood cohesion was associated with being more likely to accurately perceive the objective proximity to a park. It may be that knowledge of local facilities and routes to and from those facilities is more closely aligned to reality for those who feel more socially connected to their local area, through greater use of local resources. It is also possible that those with better local knowledge through resource utilisation develop a greater sense of community. It has previously been demonstrated that the retail environment can provide a valuable social space for interactions with others [45], and has great ‘linking value’ for social connectivity needs [29]. Longitudinal studies would contribute to understanding the temporal ordering of this association.

Greater physical functioning capacity was associated in this study with a lesser likelihood of discordance between perceived and objective measures of the neighbourhood food environment. A potential explanation for this is the method used in this study to convert walking time to perceived distance. Those with lesser physical ability may accurately perceive a greater walking time to a given destination than those with greater physical ability. In converting walking time to distance, this difference in walking time according to physical ability was not accounted for. Instead, we used an average adult walking speed to convert walking time to distances. The finding that physical activity was not a consistent correlate of inaccurate perceptions of distance to FVR, when included in the same models as physical functioning, may suggest that being able to physically get out and about, for example taking a short walk around the neighbourhood, is more important than how much one gets out and about.

In contrast to physical functioning, respondents with better mental well-being were more likely to overestimate distances to FVR. These associations were independent of age and gender, sense of community and perceptions of crime and safety, which may have confounded an association between mental well-being and the discordance between perceived and objective neighbourhood resource distance. It is possible that those reporting positive mental health might be more likely to experience positive feelings, which is known to be associated with heuristic processing [23]. This may have led to biased estimates of distances and therefore discordance between the perceived and objective distances. However, it is unclear why positive mental well-being, or the use of heuristics would lead to overestimations of distance. Without longitudinal data, it is difficult to establish whether mental well-being does in fact predict perceived-objective discordance in relation to FVR access.

Greater objectively-assessed distance to the nearest greengrocer was negatively associated with overestimating the distance to the nearest FVR. McCormack and colleagues [3] found that study participants in a sample also drawn from Adelaide, South Australia, tended to overestimate distances to destinations closer to home, but tended to underestimate distances to destinations located further from home, which may explain the results found in this study. It may also be possible that these results can be attributed to the way the distance measures were expressed. The perceived distance categories increase in size as distance becomes greater (i.e., shorter distances are in 5-min walking time categories (e.g., 1–5 min walk), whereas longer distances are expressed in 10-min walking categories (e.g., 11–20 min walk). McCormack and colleagues [3] reported that the greatest correspondence (concordance) between perceived and objective distances was found for destinations located within 750 to 1500 m of residents’ homes. Our findings concur: greater objective distance to the nearest FVR was associated with a lower likelihood of discordance between perceived and objective distances to the nearest FVR. While the study by McCormack and colleagues [3] utilised a population-based sample drawn from one high-walkable and one low-walkable neighbourhood, the final analytic sample was very small (*n* = 86). The present study had a larger population-based sample, but the sample was not intentionally drawn from neighbourhoods varying in built environment characteristics. Future studies to examine the effect of objective distances to destinations on residents’ perceptions, and specifically misperceptions of distance and availability utilising large population-based, spatially-derived samples would further contribute to this body of knowledge.

A considerable strength of this study was the inclusion of a range of neighbourhood-related characteristics, as well as individual factors, in evaluating correlates of discordance between perceived and objective neighbourhood distances in a large population-based sample. However, several limitations apply. An obvious shortcoming of this study is the cross-sectional nature of the associations evaluated. Further evaluation of prospective associations demonstrating the factors which temporally predict inaccurate perceptions would contribute stronger evidence regarding the factors associated with perceptions discordant with objective measures of environment. Additionally, measures of walkability were not included in the present analysis but are highly relevant, particularly in relation to perceived distance to resources and the influence of locomotion-based knowledge of the neighbourhood on such perceptions [46]. Future research might explore how objectively assessed walkability influences perceptions of distance to resources. Lastly, the association between food purchasing behaviour and overestimated distances to food retailers could not be assessed in the present study, as data on food purchasing behaviour was not captured in the present study. Future research exploring local resource utilisation in relation to perceived and objective access to resources is required to better understand the factors that shape perceptions of neighbourhood features, and thus related health behaviours and outcomes.

## 5. Conclusions

The findings of this study demonstrated that, beyond individual characteristics, features of neighbourhoods, including feeling a sense of community with others in the neighbourhood, were related to an overestimation of distance between perceived and objective distances to grocery retailers. This study highlights the importance of conceptualising resident perceptions of neighbourhood attributes as distinct from objective measures, given that the two types of measure are not concordant. This study also highlights the importance of understanding the factors which shape perceptual measures of the local neighbourhood environment. Treatment of perceived and objective measures of environment as distinct constructs in research will enable more appropriate implications to be drawn from the research regarding the use of the findings for interventions.

Knowledge of individual and neighbourhood factors that predict resident perceptions of the neighbourhood food environment may benefit interventions to improve perceptions of access to local healthful food resources, with the ultimate aim of increasing fruit and vegetable intake for health. For example, population-based public health interventions related to improving fruit and vegetable consumption for health benefit may include local neighbourhood-based strategies involving food retailers designed to promote and improve perceptions of the local food environment and develop residents’ sense of community. Further understanding of the relationships between perceived and objective measures of other elements of the neighbourhood environment would enhance current knowledge in this field and would contribute to the utility of research findings for population health interventions.

## Figures and Tables

**Table 1:** Individual and area characteristics of the sample (*n* = 1305).

**Individual Characteristics**	**Mean (SD) or *n* (%)**
Age (years)	55.3 (14.0)
Gender (*n* (%))	
Male	617 (47.2%)
Female	688 (52.7%)
Education level (*n* (%))	
Less than bachelor’s degree	1122 (86.0%)
Bachelor’s degree or higher	183 (14.0%)
Annual household income (AUD$) (*n* (%))	
Less than $20,001	301 (23.1%)
$20,001 to $60,000	625 (47.9%)
More than $60,000	379 (29.0%)
Duration at current residence (years)	19.7 (13.4)
Fruit & vegetable intake (number of serves per day)	4.2 (1.9)
Physical activity score (total energy expenditure (METS))	1720.3 (3082.2)
Nearest fruit/vegetable retailers (FVR): Perceived distance overestimated objective distance (*n* (%))	484 (47.5%)
Nearest FVR: Perceived distance matched objective distance (*n* (%))	535 (52.5%)
**Area Characteristics**	**Mean (SD)**
Area-level median weekly household income (AUD$)	858.6 (198.3)
Distance to the nearest FVR (metres)	1164.0 (828.1)

**Table 2:** Agreement between objective and perceived distances to the nearest type of FVR (*n* = 1305).

	Weighted κ (95% CI)	Average Weighted κ (95% CI)
**Distance to nearest FVR**		
Supermarket	0.20 (0.16–0.23)	0.22 (0.18–0.26)
Greengrocer	0.24 (0.21–0.28)	

**Table 3:** Associations between individual and neighbourhood factors and overestimation of distance to FVR (*n* = 1019).

	Overestimation ^a^ of Distance to FVR
	OR (95% CI)
Age (10-year increase)	1.10 (0.96 1.26)
Gender	
Male	0.89 (0.66, 1.19)
Female	1.00
Annual household income (AUD)	
Less than $20,001	1.00
$20,001 to $60,000	0.69 (0.48, 0.98) *
$60,001 or more	0.45 (0.28, 0.71) **
Educational attainment	
Less than bachelor’s degree	1.05 (0.68, 1.61)
Bachelor’s degree or higher	1.00
Years lived in current residence (5-year increase)	0.99 (0.93, 1.05)
Physical activity (1000 MET increase)	0.99 (0.95, 1.03)
Fruit & vegetable intake (1 serve/day increase)	0.94 (0.87, 1.01)
Physical functioning (10-point increase)	0.84 (0.78, 0.90) **
Mental well-being (10-point increase)	1.15 (1.04, 1.26) **
Sense of community (0 = Strongly Disagree to 5 = Strongly Agree) (1-point increase)	0.82 (0.72, 0.94) *
Perceived crime in the local area (1 unit increase in factor score)	1.09 (0.93, 1.27)
Area median weekly income ($100 increase)	1.09 (0.97, 1.22)
Area % with bachelor’s degree (10% increase)	0.86 (0.61, 1.22)
Objective distance to the nearest supermarket (400 m increase)	0.99 (0.92, 1.06)
Objective distance to the nearest greengrocer (400 m increase)	0.82 (0.74, 0.91) **

OR: Odds Ratio; 95% CI: 95% Confidence Interval; FVR: Fruit and Vegetable Retailers. ^a^ Compared to accurate perceptions. * *p* < 0.05; ** *p* < 0.01.
